# Association of the Maternal *MTHFR C677T* Polymorphism with Susceptibility to Neural Tube Defects in Offsprings: Evidence from 25 Case-Control Studies

**DOI:** 10.1371/journal.pone.0041689

**Published:** 2012-10-03

**Authors:** Lifeng Yan, Lin Zhao, Yan Long, Peng Zou, Guixiang Ji, Aihua Gu, Peng Zhao

**Affiliations:** 1 State Key Laboratory of Reproductive Medicine, Institute of Toxicology, Nanjing Medical University, Nanjing, China; 2 Key Laboratory of Modern Toxicology of Ministry of Education, School of Public Health, Nanjing Medical University, Nanjing, China; 3 Department of Neurosurgery, The First Affiliated Hospital, Nanjing Medical University, Nanjing, China; 4 Department of Pharmacology, China Pharmaceutical University, Nanjing, China; 5 Nanjing Institute of Environmental Sciences/Key Laboratory of Pesticide Environmental Assessment and Pollution Control, Ministry of Environmental Protection, Nanjing, China; University of Texas School of Public Health, United States of America

## Abstract

**Background:**

Methylenetetrahydrofolate reductase (*MTHFR*) is a critical enzyme in folate metabolism and is involved in DNA methylation, DNA synthesis, and DNA repair. In addition, it is a possible risk factor in neural tube defects (NTDs). The association of the *C677T* polymorphism in the *MTHFR* gene and NTD susceptibility has been widely demonstrated, but the results remain inconclusive. In this study, we performed a meta-analysis with 2429 cases and 3570 controls to investigate the effect of the *MTHFR C677T* polymorphism on NTDs.

**Methods:**

An electronic search of PubMed and Embase database for papers on the *MTHFR C677T* polymorphism and NTD risk was performed. All data were analysed with STATA (version 11). Odds ratios (ORs) with 95% confidence intervals (CIs) were estimated to assess the association. Sensitivity analysis, test of heterogeneity, cumulative meta-analysis, and assessment of bias were performed in our meta-analysis.

**Results:**

A significant association between the *MTHFR C677T* polymorphism and NTD susceptibility was revealed in our meta-analysis ( TT versus CC: OR  = 2.022, 95% CI: 1.508, 2.712; CT+TT versus CC: OR  = 1.303, 95% CI: 1.089, 1.558; TT versus CC+CT: OR  = 1.716, 95% CI: 1.448, 2.033; 2TT+CT versus 2CC+CT: OR  = 1.330, 95% CI: 1.160, 1.525). Moreover, an increased NTD risk was found after stratification of the *MTHFR C677T* variant data by ethnicity and source of controls.

**Conclusion:**

The results suggested the maternal *MTHFR C677T* polymorphism is a genetic risk factor for NTDs. Further functional studies to investigate folate-related gene polymorphisms, periconceptional multivitamin supplements, complex interactions, and the development of NTDs are warranted.

## Introduction

Neural tube defects (NTDs) are a group of severe congenital malformations with an average worldwide birth prevalence of 1 in 500 [1], occurring due to incomplete closure of the neural tube between days 22 and 26 (somite stage 10–12) during embryo development [2]. These birth defects can cause lifelong disability or death.

Although the cause of NTDs is still poorly understood, accumulated evidence has suggested that genetic and/or environmental factors may contribute to NTD aetiology. Among these factors, maternal nutritional status is a key determinant of pregnancy outcome, and attention has been focused on folic acid, a water-soluble B vitamin that acts as a cofactor in one-carbon transfer reactions and plays a central role in DNA methylation, synthesis, and repair [3], [4]. It has been shown that the occurrence and recurrence risk of NTDs is reduced by 50–70% with folic acid supplementation during the periconceptional period [5]. However, the underlying mechanisms by which folic acid protects against NTDs are still unknown. In addition, it is not known why some women who take folic acid supplements during the periconceptional period still have offspring with NTDs [6]. Therefore, candidate genes that encode enzymes involved in folate metabolism or receptors involved in folate transport have been analysed.

**Figure 1:**
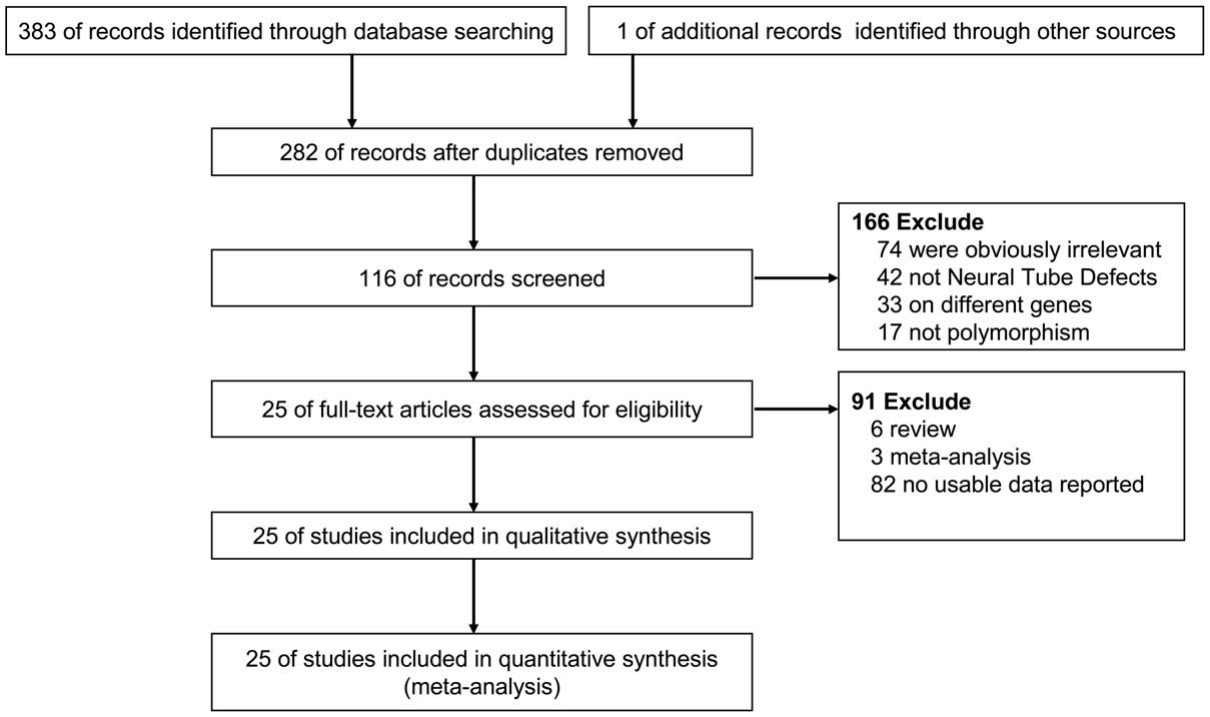
The studies inclusion and exclusion procedures.

The enzyme *MTHFR* plays a key role in the folate metabolism pathway and regulates the intracellular folate pool for synthesis and methylation of DNA [7], [8]. The *MTHFR* gene is located at chromosome 1p36.3 and is 2.2 kb in length with a total of 11 exons [9]. Several single nucleotide polymorphisms in the *MTHFR* gene have been characterised, with the *C677T* polymorphism as the most important and best studied [10]. The C-to-T transition at nucleotide 677 in exon 4 is a point mutation that converts a cytosine (C) to a thymine (T), resulting in an amino acid substitution of alanine to valine [11], which can be detected functionally because it decreases the thermal stability of this enzyme during in vitro incubation of cell extracts at 46°C for 5 min [12]. This mutation reduces enzyme activity, decreases concentration of folate in the serum, plasma, and red blood cells and increases total plasma homocysteine concentrations [13], which explains a substantial part of the observed elevated plasma homocysteine levels in mothers with NTD-affected offspring.

**Table 1:** Characteristics of the association studies on maternal MTHFRC677T polymorphism and the risk of Neural tube defects (NTDs).

ID	first author	year	Source of control	Region	Ethnicity	total	Case genetypes CC/CT/TT	Control genetypes CC/CT/TT	HWE
1	Arbour L [17]	2002	mixed	Canada	Caucasian	175	32/31/11	52/38/11	0.319
2	Candito M [18]	2008	HB	France	Caucasian	138	25/40/12	26/29/6	0.610
3	Ceyhan ST [19]	2008	HB	Turkey	Caucasian	64	9/14/6	20/12/3	0.544
4	Christensen B [20]	1999	HB	Canada	Caucasian	152	24/27/11	44/36/10	0.526
5	Dalal A [21]	2007	PB	India	Caucasian	143	56/21/6	45/12/3	0.095
6	Deb R [22]	2011	PB	India	Caucasian	333	80/25/6	149/64/9	0.524
7	Félix TM [23]	2004	HB	Brazil	Caucasian	85	19/15/7	16/22/6	0.718
8	Godbole K [24]	2011	HB	India	Caucasian	989	238/62/5	521/158/5	0.059
9	Houcher B [25]	2009	HB	Algeria	African	174	35/42/15	33/35/14	0.375
10	Lacasana M [26]	2012	HB	Mexico	Mixed	189	11/45/42	20/49/22	0.460
11	Li K [27]	2000	HB	China	Asian	51	1/17/9	5/16/3	0.093
12	Lucock M [28]	2000	PB	UK	Caucasian	50	8/9/2	11/17/3	0.330
13	Martínez de Villarreal LE [29]	2001	PB	Mexico	Mixed	69	11/12/15	12/16/3	0.479
14	Molloy AM [30]	1998	HB	Ireland	Caucasian	343	34/35/13	119/121/21	0.200
15	Munoz JB [31]	2007	HB	Mexico	Mixed	230	14/54/50	25/57/30	0.833
16	Naushad SM [32]	2010	PB	India	Caucasian	130	33/11/6	64/16/0	0.320
17	Parle-McDermott A [33]	2003	HB	Ireland	Caucasian	529	102/138/34	126/103/26	0.469
18	Perez AB [34]	2003	HB	Brazil	Mixed	257	67/55/9	70/54/2	0.019*
19	Relton CL [35]	2004	HB	UK	Caucasian	698	86/78/22	191/254/67	0.222
20	Relton CL [36]	2004	HB	UK	Caucasian	251	31/36/15	66/88/15	0.058
21	Shang Y [37]	2008	HB	China	Asian	118	14/20/4	25/38/17	0.718
22	Shields DC [38]	1999	HB	Ireland	Caucasian	460	80/108/30	114/108/20	0.426
23	Ubbink JB [39]	1999	PB	Africa	African	107	42/11/0	43/11/0	0.405
24	Wang F [40]	2008	HB	China	Asian	198	14/50/35	34/48/17	0.990
25	Yu J [41]	2000	PB	China	Asian	66	2/25/15	5/16/3	0.093

* P<0.05.

In the past decade, studies have investigated the association between the *C677T MTHFR* polymorphism and NTD susceptibility. However, these studies have failed to yield a consistent conclusion. Therefore, we performed a meta-analysis of all studies published until January 2012 to explore this inconsistency and to investigate the association between the maternal *MTHFR C677T* polymorphism and risk of NTDs.

**Figure 2:**
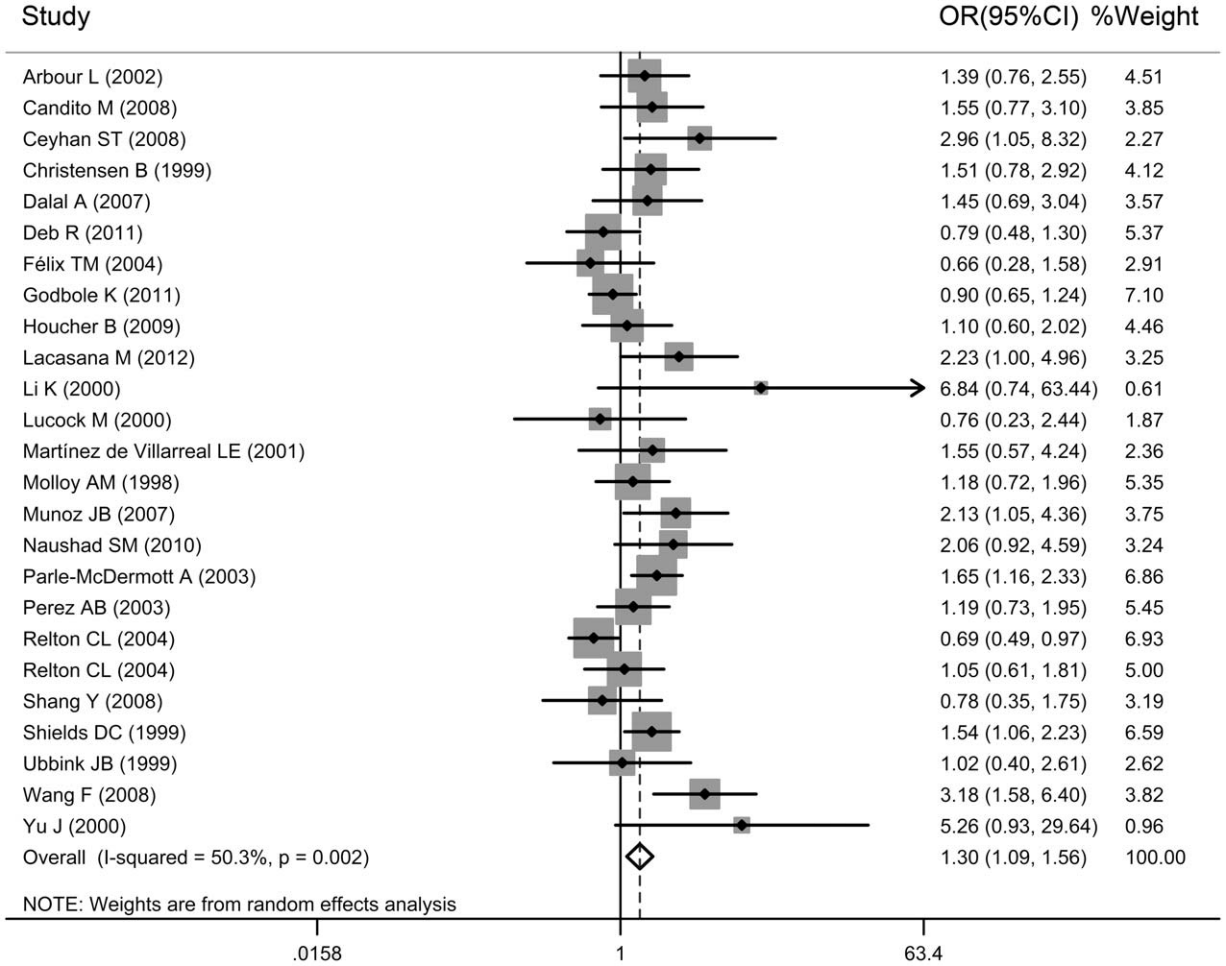
Forest plot of NTD risk associated with the maternal *MTHFR C677T* polymorphism (TT+CT vs. CC) in overall populations.

## Materials and Methods

### Study eligibility

Potentially relevant reports were selected by searching Embase and PubMed (the last search update was performed on January 10, 2012) using the main search terms “methylene-tetrahydrofolate reductase,” “*MTHFR*,” and “neural tube defects,” “NTD”. All studies were published in English or in the Chinese language, and were human studies only. The related reference articles were searched to identify other relevant publications. Unpublished data and further information were also obtained from the authors.

**Table 2:** Summary of comparisons for maternal MTHFRC677T polymorphism and risk of Neural tube defects (NTDs).

Contrast	Variables	Comparisons	OR	95% CI	P	P*_heterogeneity_
TT vs CC	Overall	24	**2.022**	**1.508–2.712**	**<0.001**	0.006
	Study design					
	PB	6	**2.756**	**1.528–4.970**	**0.001**	0.112
	HB	17	**1.951**	**1.404–2.713**	**<0.001**	0.006
	Mixed	1	1.625	0.632–4.179	0.314	-
	Ethnicity					
	Asian	4	3.750	0.732–19.217	0.113	0.003
	Caucasian	15	**1.596**	**1.271–2.005**	**<0.001**	0.280
	African	1	1.010	0.423–2.411	0.982	-
	Mixed	4	**3.595**	**2.139–6.042**	**<0.001**	0.888
CT vs CC	Overall	25	1.154	0.982–1.357	0.083	0.053
	Study design					
	PB	7	0.991	0.717–1.370	0.955	0.525
	HB	17	1.188	0.973–1.451	0.014	0.017
	Mixed	1	1.326	0.694–2.532	0.393	-
	Ethnicity					
	Asian	4	**1.933**	**1.167–3.202**	**0.010**	0.198
	Caucasian	15	1.080	0.886–1.316	0.445	0.039
	African	2	1.095	0.641–1.872	0.740	0.864
	Mixed	4	1.248	0.877–1.775	0.218	0.566
CT+TT vs CC	Overall	25	**1.303**	**1.089–1.558**	**0.004**	0.002
	Study design					
	PB	7	1.174	0.867–1.588	0.299	0.210
	HB	17	**1.318**	**1.060–1.639**	**0.013**	0.001
	Mixed	1	1.393	0.762–2.546	0.282	-
	Ethnicity					
	Asian	4	2.455	0.886–6.803	0.084	0.026
	Caucasian	15	1.186	0.968–1.454	0.100	0.011
	African	2	1.075	0.644–1.792	0.783	0.904
	Mixed	4	**1.576**	**1.125–2.208**	**0.008**	0.450
TT vs CT+CC	Overall	24	**1.716**	**1.448–2.033**	**<0.001**	0.134
	Study design					
	PB	6	**2.818**	**1.62–4.882**	**<0.001**	0.214
	HB	17	**1.631**	**1.359–1.958**	**<0.001**	0.147
	Mixed	1	1.429	0.583–3.498	0.435	-
	Ethnicity					
	Asian	4	1.949	0.752–5.056	0.170	0.033
	Caucasian	15	**1.524**	**1.227–1.893**	**<0.001**	0.632
	African	1	0.946	0.426–2.102	0.892	-
	Mixed	4	**2.514**	**1.720–3.676**	**<0.001**	0.412
2TT+CT vs 2CC +CT	Overall	25	**1.330**	**1.160–1.525**	**<0.001**	0.001
	Study design					
	PB	7	**1.462**	**1.006–2.124**	**0.047**	0.045
	HB	17	**1.305**	**1.116–1.525**	**0.001**	0.001
	Mixed	1	1.320	0.840–2.074	0.228	-
	Ethnicity					
	Asian	4	1.633	0.908–2.939	0.102	0.009
	Caucasian	15	**1.223**	**1.049–1.427**	**0.010**	0.013
	African	2	1.029	0.698–1.516	0.886	0.985
	Mixed	4	**1.653**	**1.331–2.052**	**<0.001**	0.470

* : Random-effects model was used when P value for heterogeneity test<0.10; otherwise, fix-effects model was used.

### Validity assessment

Potential studies were selected following inclusion criteria: 1) *MTHFR C677T* polymorphism and NTDs; 2) human case-control design; 3) sufficient maternal genotype data for estimating an odds ratio (OR) with a 95% confidence interval (CI); and 4) published in English or Chinese. The criteria for the exclusion of studies are as follows: 1) not related to the *MTHFR C677T* polymorphism and NTDs; 2) not a primary case-control study; 3) no usable or sufficient maternal genotype data reported: and 4) controls are not mothers with at least one healthy birth.

**Figure 3:**
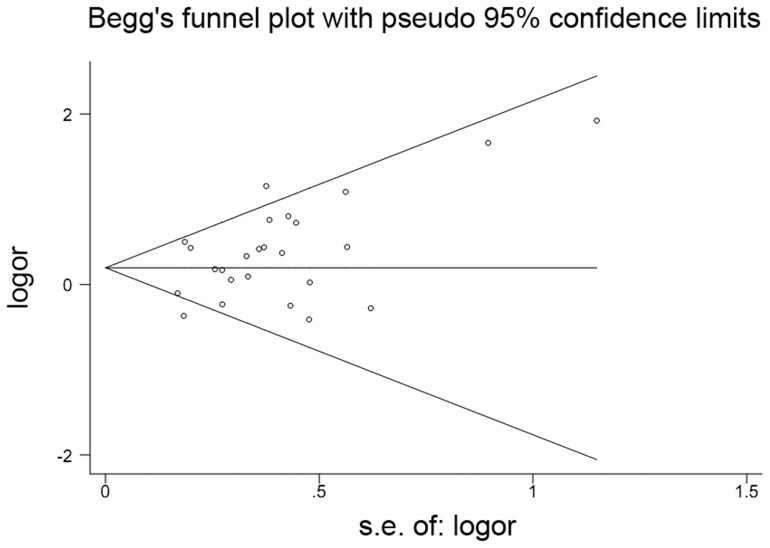
Begg's Funnel plot of NTD risk associated with the maternal *MTHFR C677T* polymorphism (TT vs. CC +CT) in overall populations. Each point represents a separate study for the indicated association.

### Data extraction

Two investigators independently extracted the data from all eligible studies using the selection criteria listed above. Any disagreement was resolved by discussion. We extracted the following information: the first author's name, year of publication, the country in which the study was conducted, the ethnicities of the individuals involved, the source of control groups (population-based or hospital-based controls or mixed), the sample size, number of cases and controls with the CC, CT, and TT genotypes.

### Data synthesis

All statistical analyses were performed using the STATA software (version 11). Two-sided *P* values less than 0.05 were considered statistically significant. For the control groups of each study, the observed genotype frequencies of the *MTHFR C677T* polymorphism were assessed for Hardy-Weinberg equilibrium.

The strength of the association between the *MTHFR C677T* polymorphism and NTD risk was evaluated by the odds ratios (ORs) with 95% confidence intervals (CIs). The pooled ORs were calculated for the homozygote comparison (TT versus CC), heterozygote comparison (CT versus CC), dominant model (CT + TT versus CC), recessive model (TT versus CT + CC), and an additive model (2TT + CT versus 2CC + CT). Subgroup analyses were performed based on the source of controls and ethnicity if the data permitted.

The evaluation of the meta-analysis results included a test for heterogeneity, an analysis of the sensitivity, and an examination for bias. The chi-squared test-based Q-statistic was calculated to test the heterogeneity between studies and detect the source of heterogeneity by ethnicity, publication year, control source, and sample size. The model used for the analysis of the pooled ORs depends on the *P* value. If the heterogeneity test result was *P*<0.1, the pooled ORs were analysed using the random-effects model (the DerSimonian and Laird method) [14]; otherwise, the fixed-effects model was used (the Mantel-Haenszel method) [15]. Additionally, sensitivity analyses were performed after sequential removal of each study. Finally, the Begg's funnel plot and Egger's test were performed to statistically analyse the publication bias [16].

## Results

### Study characteristics

We included 25 eligible studies [17]–[41] in our meta-analysis. The studies contained data on 5999 mothers (2429 case mothers and 3570 control mothers) who had an *MTHFR C677T* polymorphism in the case-control design. The characteristics of all reports on the association between the *MTHFRC677T* polymorphism and NTDs are shown in Table 1. In our meta-analysis, 7 studies were population-based controls, 17 studies were hospital-based controls, 1 did not provide detailed information regarding the source of the controls were mixed; 4 studies included Asian populations, 15 studies were Caucasian population, 2 studies were African population, and 4 studies were mixed population (white and non-white). The genotype distributions in the controls for all studies were consistent with Hardy-Weinberg equilibrium, except for the controls in a study by Perez et al.[34]. Figure 1 shows the study selection procedure.

### Evidence synthesis

In our meta-analysis the CC genotype was used as the reference group. The maternal *MTHFRC677T* polymorphism showed pooled odds ratios for homozygote comparison (TT versus CC: OR  = 2.022, 95% CI: 1.508, 2.712, *P*<0.001), for dominant model comparison (CT+TT versus CC: OR  = 1.303, 95% CI: 1.089, 1.558, *P* = 0.004), for recessive model comparison (TT versus CC+CT: OR  = 1.716, 95% CI: 1.448, 2.033, *P*<0.001), and for additive model comparison (2TT+CT versus 2CC+CT: OR  = 1.330, 95% CI: 1.160, 1.525, *P*<0.001). Overall, there was a significant association between the maternal *MTHFR C677T* polymorphism and NTDs. The forest plot is shown in Figure 2.

### Subgroup analysis

We also performed subgroup analysis stratified by ethnicity and study design. We found that the variant genotypes were associated with a significantly increased NTD risk in Asian, Caucasian and mixed populations. In heterozygote comparison ( CT versus CC), the pooled odds ratio was 1.933 (95%CI: 1.167, 3.202, *P* = 0.010) for Asian population. The pooled odds ratios were 1.524 (95% CI: 1.227, 1.893, *P*<0.001), and 2.514 (95% CI: 1.720, 3.676, *P*<0.001) for Caucasian and mixed populations, respectively, under the recessive model. However, we did not find an association between the *C677T* polymorphism and NTD risk in African groups in any genetic models. The meta-analysis results for the other genetic models are listed in Table 2.

Significantly increased risks were also found in the subgroup analysis stratified by the source of the controls. The pooled odds ratios were 2.756 (95% CI: 1.528, 4.970, *P* = 0.001) in the population-based control subgroups and 1.951 (95% CI: 1.404, 2.713, *P*<0.001) in the hospital-based control subgroups by homozygote comparison. The meta-analysis results for the other genetic models are listed in Table 2.

### Test for heterogeneity

There was significant heterogeneity in four genetic models: TT versus CC: *P*_heterogeneity_  = 0.006; CT versus CC: *P*_heterogeneity_  = 0.053; CT + TT versus CC: *P*_heterogeneity_  = 0.002; and 2TT + CT versus 2CC + CT: *P*_heterogeneity_ <0.001). Data are listed in Table 2. We assessed the source of heterogeneity by ethnicity, publication year, control source, and sample size. However, we did not observe any sources that contribute to the substantial heterogeneity.

### Sensitivity analysis and cumulative meta-analysis

Sensitivity analyses were conducted to ascertain the primary origin of the heterogeneity. Two independent studies by Relton CL[35] and Wang F[40] affected the heterogeneity in maternal case-control studies. The heterogeneity was effectively decreased by the exclusion of the two studies: Heterogeneity chi-squared  = 48.33, *P*_heterogeneity_  = 0.002 and heterogeneity chi-squared  = 29.94, *P*_heterogeneity_  = 0.120, before and after removal, respectively. Furthermore, no single study qualitatively changed the pooled ORs, suggesting that the results of this meta-analysis were stable. In the cumulative meta-analysis, the pooled ORs tended to be stable, and the associations tended towards significant associations with the accumulation of more data over time.

### Publication bias

Funnel plots were generated to assess publication bias. Egger's test was performed to statistically evaluate funnel plot symmetry. The results showed no evidence of publication bias: *P* = 0.034, 95% CI: 0.125, 2.824 (Figure 3).

## Discussion

The folate metabolism pathway plays an important role in DNA methylation, DNA synthesis, cell division, and tissue growth, especially in the rapidly developing cells [42]. Thus, a defective folate metabolism could result in an impaired DNA synthesis or DNA methylation involved in the neurulation process. *MTHFR* is a key enzyme in the folate metabolism pathway. Although several single nucleotide polymorphisms (SNPs) in the *MTHFR* gene have been characterised, the *C677T* polymorphism is a widely described mutation. Heterozygotes (CT) for the polymorphism have 65% of the normal enzyme activity and 10% lower red blood cell folate level; patients with the homozygous variant (TT) have only 30% of normal enzyme activity and 18% lower red blood cell folate levels [43], [44]. Additionally, individuals with the TT variant also have lowered plasma folate and vitamin B_12_ levels and increased homocysteine levels [45], [46]. Overall, due to its potential role in decreasing *MTHFR* activity, causing high plasma homocysteine and low plasma folate levels, it contributes to NTDs [47]. Despite the potential implication of *MTHFR C677T* in the pathogenesis of NTDs [18]–[21], [25]–[27], [29]–[34], [37], [38], [40], [41], the association between the *MTHFR C677T* polymorphism and NTDs remains unclear.

Our meta-analysis, which included 2429 cases and 3570 controls, explored the associations between the maternal *MTHFR* C667T polymorphism and susceptibility to NTDs. Overall, we found that mothers with the homozygous TT genotype showed a significantly increased NTD risk compared with homozygous CC genotype carriers (with pooled odds ratio 2.022; 95% CI: 1.508, 2.712; *P*<0.001). Our results were consistent with a previous report [48]that showed an overall odds ratio of 2.04 (TT versus CC: 95% CI: 1.49, 2.81).

In subgroup analysis stratified by ethnicity, we found that the variant genotypes were associated with a significantly increased NTD risk in Asian, Caucasian and mixed populations. However, we did not found this association in African groups in any genetic model, possibly due to the limited studies and a small sample size. Moreover, the pooled odds ratios of mixed populations were higher than those of Caucasian populations and the overall populations in mothers (TT versus CC: OR = 3.595, 1.596, and 2.022, respectively). Many factors may contribute to the finding that the same polymorphism affects different ethnic populations to a different extent. First, the frequency of the T-allele varies in different ethnicities with different genetic backgrounds [49]. Second, different populations may have different dietary patterns, such as intake of folic acid, vitamin B_12_, and vitamin B_6_, some of which may affect NTD development. Finally, analysis of the data from the various ethnic groups might eliminate some bias caused by language because only papers written in English or Chinese were included. Thus, large-scale studies should be performed to validate ethnic differences in the effect of this functional polymorphism on NTD risk.

When stratified by study design, significantly increased risks were also found in both population-based and hospital-based studies. Nevertheless, population-based studies have a higher risk than hospital-based studies. Hospital-based studies usually have a high risk of producing unreliable results because hospital-based controls may not always accurately represent the general population, especially when the genotypes under investigation are expected to affect disease conditions that might be observed in the hospital-based controls [50]. Thus, in genetic association studies, the selection of controls and matching status should be carefully considered. To reduce the bias, well-designed, population-based studies should be performed to explore the association.

Because heterogeneity is a potential problem when interpreting the results of all meta-analyses, we detected the source of heterogeneity by ethnicity, publication year, control source, and sample size and found that none substantially contributed to the heterogeneity. One possible reason might be the matching status.

It is assumed that *MTHFR* genetic polymorphisms play an important role in the development of NTDs; however, only 13% of NTDs were attributed to the *MTHFR C677T* mutation [51], suggesting that the *MTHFR C677T* polymorphism alone cannot be responsible for NTDs. Thus, potential gene-gene, maternal-foetal, genetic-nutritional interactions [48], and other SNPs in the *MTHFR* gene may have an association with NTD risk.

### 

#### 1) Gene-gene interactions

Folate metabolism is complex and involves several regulatory mechanisms. Genetic variations affecting protein function at any step may alter the balance of metabolites, and gene-gene interactions [52]. The combination of *MTHFR* and cystathione-β-synthase (CBS) mutations was reported to have a fivefold increase in the risk for spina bifida compared with each variant alone [53], indicating the presence of gene-gene interactions.

#### 2) Maternal-foetal interactions

Maternal variant genotypes were associated with NTD risk, indicating possible maternal-foetal interactions. Using family-based approaches, researchers have found that the OR increased to 4.1 (95% CI: 1.5, 11.1) if the mother had a TT genotype and her child a CT genotype and to 6.1 (95% CI: 1.0, 35.5) if both the mother and her child had TT genotypes [54].

#### 3) Genetic-nutritional interactions

The combination of *MTHFR* mutations and low folate concentrations could lead to a hypomethylation of homocysteine to methionine, enhancing the impairment of folate metabolism and increasing the risk for NTDs [1], suggesting a strong genetic-nutritional interaction. This interaction was found in a previous study that showed the combination of *MTHFR* TT genotype and RBC folate level in the lowest quartile conferred an odds ratio of 13.43 (95% CI: 2.49, 72.33) for an NTD case and an odds ratio of 3.28 (95% CI: 0.84, 12.85) for having offspring with NTDs [20].

Although these studies were hampered by small sample sizes, they illustrate the existence of potential interactions. Thus, further large-scale studies focusing on these complex interactions with NTD risk should be performed.

#### 4) Other SNPs in the *MTHFR* gene

Some researchers have demonstrated that other SNPs in *MTHFR* gene showed increased NTD risks, such as *A1298C* [55], [56], *C116T* [57], *G1793A* [57], and were linkage disequilibrium with *C677T* polymorphism. All these suggest these SNPs can be additional genetic factors for NTDs.

### Limitations

Several potential limitations of this meta-analysis should be discussed. 1) Although the funnel plot and Egger's test showed no publication bias, selection bias may have occurred because only studies in English or Chinese were selected. 2) Our results were based on unadjusted estimates due to the absence of available information, such as supplementation of folic acid, maternal use of drugs, and other factors that are associated with NTD risk. 3) We did not consider the foetal *MTHFR C677T* polymorphisms due to the limited data. Despite the limitations listed above, our meta-analysis has some clear advantages. 1) The well-designed search and selection method significantly increased the statistical power of this meta-analysis. 2) No publication bias was detected, indicating that our pooled results are likely to be reliable.

In conclusion, our meta-analysis provided evidence that the *MTHFR C677T* polymorphism is a genetic risk factor for NTDs. Because NTDs are the most common congenital malformations worldwide and have a multifactorial aetiology, various genetic and environmental factors as well as their complex interactions implicated in the pathogenesis should be taken into account. Further functional studies to investigate folate-related gene polymorphisms, periconceptional multivitamin supplements, complex interactions and their role in development of NTDs are warranted.

## References

[1] van der Put NM, van Straaten HW, Trijbels FJ, Blom HJ (2001) Folate, homocysteine and neural tube defects: an overview. Exp Biol Med (Maywood) 226: 243–270. 11368417 10.1177/153537020122600402

[2] Koch MC, Stegmann K, Ziegler A, Schroter B, Ermert A (1998) Evaluation of the MTHFR C677T allele and the MTHFR gene locus in a German spina bifida population. Eur J Pediatr 157: 487–492. 9667406 10.1007/s004310050860

[3] Hosseini M, Houshmand M, Ebrahimi A (2011) MTHFR polymorphisms and breast cancer risk. Arch Med Sci 7: 134–137. 22291746 10.5114/aoms.2011.20618 PMC3258688

[4] Zeisel SH (2009) Importance of methyl donors during reproduction. Am J Clin Nutr 89: 673S–677S. 19116320 10.3945/ajcn.2008.26811D PMC2628952

[5] Speer MC, Worley G, Mackey JF, Melvin E, Oakes WJ, et al (1997) The thermolabile variant of methylenetetrahydrofolate reductase (MTHFR) is not a major risk factor for neural tube defect in American Caucasians. The NTD Collaborative Group. Neurogenetics 1: 149–150. 10732818 10.1007/s100480050022

[6] Marini NJ, Hoffmann TJ, Lammer EJ, Hardin J, Lazaruk K, et al (2011) A genetic signature of spina bifida risk from pathway-informed comprehensive gene-variant analysis. PLoS One 6: e28408. 22140583 10.1371/journal.pone.0028408 PMC3227667

[7] Das PM, Singal R (2004) DNA methylation and cancer. J Clin Oncol 22: 4632–4642. 15542813 10.1200/JCO.2004.07.151

[8] Ueland PM, Hustad S, Schneede J, Refsum H, Vollset SE (2001) Biological and clinical implications of the MTHFR C677T polymorphism. Trends Pharmacol Sci 22: 195–201. 11282420 10.1016/s0165-6147(00)01675-8

[9] Goyette P, Sumner JS, Milos R, Duncan AM, Rosenblatt DS, et al (1994) Human methylenetetrahydrofolate reductase: isolation of cDNA, mapping and mutation identification. Nat Genet 7: 195–200. 7920641 10.1038/ng0694-195

[10] Sameer AS, Shah ZA, Nissar S, Mudassar S, Siddiqi MA (2011) Risk of colorectal cancer associated with the methylenetetrahydrofolate reductase (MTHFR) C677T polymorphism in the Kashmiri population. Genet Mol Res 10: 1200–1210. 21732284 10.4238/vol10-2gmr1067

[11] Sharp L, Little J (2004) Polymorphisms in genes involved in folate metabolism and colorectal neoplasia: a HuGE review. Am J Epidemiol 159: 423–443. 14977639 10.1093/aje/kwh066

[12] Kang SS, Wong PW (1996) Genetic and nongenetic factors for moderate hyperhomocyst(e)inemia. Atherosclerosis 119: 135–138. 8808490 10.1016/0021-9150(95)05648-3

[13] van der Put NM, Blom HJ (2000) Neural tube defects and a disturbed folate dependent homocysteine metabolism. Eur J Obstet Gynecol Reprod Biol 92: 57–61. 10986435 10.1016/s0301-2115(00)00426-7

[14] DerSimonian R, Laird N (1986) Meta-analysis in clinical trials. Control Clin Trials 7: 177–188. 3802833 10.1016/0197-2456(86)90046-2

[15] Mantel N, Haenszel W (1959) Statistical aspects of the analysis of data from retrospective studies of disease. J Natl Cancer Inst 22: 719–748. 13655060

[16] Egger M, Davey Smith G, Schneider M, Minder C (1997) Bias in meta-analysis detected by a simple, graphical test. BMJ 315: 629–634. 9310563 10.1136/bmj.315.7109.629 PMC2127453

[17] Arbour L, Christensen B, Delormier T, Platt R, Gilfix B, et al (2002) Spina bifida, folate metabolism, and dietary folate intake in a Northern Canadian aboriginal population. Int J Circumpolar Health 61: 341–351. 12546192 10.3402/ijch.v61i4.17492

[18] Candito M, Rivet R, Herbeth B, Boisson C, Rudigoz RC, et al (2008) Nutritional and genetic determinants of vitamin B and homocysteine metabolisms in neural tube defects: a multicenter case-control study. Am J Med Genet A 146A: 1128–1133. 18386810 10.1002/ajmg.a.32199

[19] Ceyhan ST, Beyan C, Bahce M, Baser I, Kaptan K, et al (2008) Thrombophilia-associated gene mutations in women with pregnancies complicated by fetal neural tube defects. Int J Gynaecol Obstet 101: 188–189. 18068170 10.1016/j.ijgo.2007.10.006

[20] Christensen B, Arbour L, Tran P, Leclerc D, Sabbaghian N, et al (1999) Genetic polymorphisms in methylenetetrahydrofolate reductase and methionine synthase, folate levels in red blood cells, and risk of neural tube defects. Am J Med Genet 84: 151–157. 10323741 10.1002/(sici)1096-8628(19990521)84:2<151::aid-ajmg12>3.0.co;2-t

[21] Dalal A, Pradhan M, Tiwari D, Behari S, Singh U, et al (2007) MTHFR 677C–>T and 1298A–>C polymorphisms: evaluation of maternal genotypic risk and association with level of neural tube defect. Gynecol Obstet Invest 63: 146–150. 17085942 10.1159/000096735

[22] Deb R, Arora J, Meitei SY, Gupta S, Verma V, et al (2011) Folate supplementation, MTHFR gene polymorphism and neural tube defects: a community based case control study in North India. Metab Brain Dis 26: 241–246. 21792640 10.1007/s11011-011-9256-8

[23] Felix TM, Leistner S, Giugliani R (2004) Metabolic effects and the methylenetetrahydrofolate reductase (MTHFR) polymorphism associated with neural tube defects in southern Brazil. Birth Defects Res A Clin Mol Teratol 70: 459–463. 15259035 10.1002/bdra.20011

[24] Godbole K, Gayathri P, Ghule S, Sasirekha BV, Kanitkar-Damle A, et al (2011) Maternal one-carbon metabolism, MTHFR and TCN2 genotypes and neural tube defects in India. Birth Defects Res A Clin Mol Teratol 91: 848–856. 21770021 10.1002/bdra.20841

[25] Houcher B, Bourouba R, Djabi F, Yilmaz E, Egin Y, et al (2009) Polymorphisms of 5,10-methylenetetrahydrofolate reductase and cystathionine beta-synthase genes as a risk factor for neural tube defects in Setif, Algeria. Pediatr Neurosurg 45: 472–477. 20160465 10.1159/000283086

[26] Lacasana M, Blanco-Munoz J, Borja-Aburto VH, Aguilar-Garduno C, Rodriguez-Barranco M, et al.. (2012) Effect on risk of anencephaly of gene-nutrient interactions between methylenetetrahydrofolate reductase C677T polymorphism and maternal folate, vitamin B12 and homocysteine profile. Public Health Nutr: 1–10. 10.1017/S136898001100334X 22230335

[27] Li K, Zheng D, Xue Y, Sun Y, Chen L, et al (2000) [The common C677T polymorphism in the methylenetetrahydrofolate reductase gene is associated with neural tube defects and preeclampsia]. Zhonghua Yi Xue Yi Chuan Xue Za Zhi 17: 76–78. 10751524

[28] Lucock M, Daskalakis I, Briggs D, Yates Z, Levene M (2000) Altered folate metabolism and disposition in mothers affected by a spina bifida pregnancy: influence of 677c–> t methylenetetrahydrofolate reductase and 2756a–> g methionine synthase genotypes. Mol Genet Metab 70: 27–44. 10833329 10.1006/mgme.2000.2994

[29] Martinez de Villarreal LE, Delgado-Enciso I, Valdez-Leal R, Ortiz-Lopez R, Rojas-Martinez A, et al (2001) Folate levels and N(5), N(10)-methylenetetrahydrofolate reductase genotype (MTHFR) in mothers of offspring with neural tube defects: a case-control study. Arch Med Res 32: 277–282. 11440783 10.1016/s0188-4409(01)00292-2

[30] Molloy AM, Mills JL, Kirke PN, Ramsbottom D, McPartlin JM, et al (1998) Low blood folates in NTD pregnancies are only partly explained by thermolabile 5,10-methylenetetrahydrofolate reductase: low folate status alone may be the critical factor. Am J Med Genet 78: 155–159. 9674907

[31] Munoz JB, Lacasana M, Cavazos RG, Borja-Aburto VH, Galaviz-Hernandez C, et al (2007) Methylenetetrahydrofolate reductase gene polymorphisms and the risk of anencephaly in Mexico. Mol Hum Reprod 13: 419–424. 17439956 10.1093/molehr/gam017

[32] Naushad SM, Devi AR (2010) Role of parental folate pathway single nucleotide polymorphisms in altering the susceptibility to neural tube defects in South India. J Perinat Med 38: 63–69. 20047525 10.1515/jpm.2009.119

[33] Parle-McDermott A, Mills JL, Kirke PN, O'Leary VB, Swanson DA, et al (2003) Analysis of the MTHFR 1298A–>C and 677C–>T polymorphisms as risk factors for neural tube defects. J Hum Genet 48: 190–193. 12730722 10.1007/s10038-003-0008-4

[34] Perez AB, D'Almeida V, Vergani N, de Oliveira AC, de Lima FT, et al (2003) Methylenetetrahydrofolate reductase (MTHFR): incidence of mutations C677T and A1298C in Brazilian population and its correlation with plasma homocysteine levels in spina bifida. Am J Med Genet A 119A: 20–25. 12707953 10.1002/ajmg.a.10059

[35] Relton CL, Wilding CS, Pearce MS, Laffling AJ, Jonas PA, et al (2004) Gene-gene interaction in folate-related genes and risk of neural tube defects in a UK population. J Med Genet 41: 256–260. 15060097 10.1136/jmg.2003.010694 PMC1735724

[36] Relton CL, Wilding CS, Laffling AJ, Jonas PA, Burgess T, et al (2004) Low erythrocyte folate status and polymorphic variation in folate-related genes are associated with risk of neural tube defect pregnancy. Mol Genet Metab 81: 273–281. 15059614 10.1016/j.ymgme.2003.12.010

[37] Shang Y, Zhao H, Niu B, Li WI, Zhou R, et al (2008) Correlation of polymorphism of MTHFRs and RFC-1 genes with neural tube defects in China. Birth Defects Res A Clin Mol Teratol 82: 3–7. 18022874 10.1002/bdra.20416

[38] Shields DC, Kirke PN, Mills JL, Ramsbottom D, Molloy AM, et al (1999) The “thermolabile” variant of methylenetetrahydrofolate reductase and neural tube defects: An evaluation of genetic risk and the relative importance of the genotypes of the embryo and the mother. Am J Hum Genet 64: 1045–1055. 10090889 10.1086/302310 PMC1377828

[39] Ubbink JB, Christianson A, Bester MJ, Van Allen MI, Venter PA, et al (1999) Folate status, homocysteine metabolism, and methylene tetrahydrofolate reductase genotype in rural South African blacks with a history of pregnancy complicated by neural tube defects. Metabolism 48: 269–274. 10024094 10.1016/s0026-0495(99)90046-x

[40] Wang F, Yang YF, Li PZ (2008) [A case-control study on the risk factors of neural tube defects in Shanxi province]. Zhonghua Liu Xing Bing Xue Za Zhi 29: 771–774. 19103110

[41] Yu J, Chen B, Zhang G, Fu S, Li P (2000) The 677 C–>T mutation in the methylenetetrahydrofolate reductase (MTHFR) gene in five Chinese ethnic groups. Hum Hered 50: 268–270. 10782023 10.1159/000022929

[42] Morrison K, Papapetrou C, Hol FA, Mariman EC, Lynch SA, et al (1998) Susceptibility to spina bifida; an association study of five candidate genes. Ann Hum Genet 62: 379–396. 10088035 10.1046/j.1469-1809.1998.6250379.x

[43] Molloy AM, Daly S, Mills JL, Kirke PN, Whitehead AS, et al (1997) Thermolabile variant of 5,10-methylenetetrahydrofolate reductase associated with low red-cell folates: implications for folate intake recommendations. Lancet 349: 1591–1593. 9174561 10.1016/S0140-6736(96)12049-3

[44] Rozen R (1997) Genetic predisposition to hyperhomocysteinemia: deficiency of methylenetetrahydrofolate reductase (MTHFR). Thromb Haemost 78: 523–526. 9198208

[45] Ma J, Stampfer MJ, Christensen B, Giovannucci E, Hunter DJ, et al (1999) A polymorphism of the methionine synthase gene: association with plasma folate, vitamin B12, homocyst(e)ine, and colorectal cancer risk. Cancer Epidemiol Biomarkers Prev 8: 825–829. 10498402

[46] Ma J, Stampfer MJ, Giovannucci E, Artigas C, Hunter DJ, et al (1997) Methylenetetrahydrofolate reductase polymorphism, dietary interactions, and risk of colorectal cancer. Cancer Res 57: 1098–1102. 9067278

[47] van der Put NM, Steegers-Theunissen RP, Frosst P, Trijbels FJ, Eskes TK, et al (1995) Mutated methylenetetrahydrofolate reductase as a risk factor for spina bifida. Lancet 346: 1070–1071. 7564788 10.1016/s0140-6736(95)91743-8

[48] Botto LD, Yang Q (2000) 5,10-Methylenetetrahydrofolate reductase gene variants and congenital anomalies: a HuGE review. Am J Epidemiol 151: 862–877. 10791559 10.1093/oxfordjournals.aje.a010290

[49] Rady PL, Szucs S, Grady J, Hudnall SD, Kellner LH, et al (2002) Genetic polymorphisms of methylenetetrahydrofolate reductase (MTHFR) and methionine synthase reductase (MTRR) in ethnic populations in Texas; a report of a novel MTHFR polymorphic site, G1793A. Am J Med Genet 107: 162–168. 11807892 10.1002/ajmg.10122

[50] Han D, Shen C, Meng X, Bai J, Chen F, et al (2012) Methionine synthase reductase A66G polymorphism contributes to tumor susceptibility: evidence from 35 case-control studies. Mol Biol Rep 39: 805–816. 21547363 10.1007/s11033-011-0802-6

[51] Boduroglu K, Alikasifoglu M, Anar B, Tuncbilek E (1999) Association of the 677C–>T mutation on the methylenetetrahydrofolate reductase gene in Turkish patients with neural tube defects. J Child Neurol 14: 159–161. 10190266 10.1177/088307389901400305

[52] Robien K, Ulrich CM (2003) 5,10-Methylenetetrahydrofolate reductase polymorphisms and leukemia risk: a HuGE minireview. Am J Epidemiol 157: 571–582. 12672676 10.1093/aje/kwg024

[53] Botto LD, Mastroiacovo P (1998) Exploring gene-gene interactions in the etiology of neural tube defects. Clin Genet 53: 456–459. 9712534 10.1111/j.1399-0004.1998.tb02594.x

[54] van der Put NM, van den Heuvel LP, Steegers-Theunissen RP, Trijbels FJ, Eskes TK, et al (1996) Decreased methylene tetrahydrofolate reductase activity due to the 677C–>T mutation in families with spina bifida offspring. J Mol Med (Berl) 74: 691–694. 8956155 10.1007/s001090050073

[55] De Marco P, Calevo MG, Moroni A, Arata L, Merello E, et al (2002) Study of MTHFR and MS polymorphisms as risk factors for NTD in the Italian population. J Hum Genet 47: 319–324. 12111380 10.1007/s100380200043

[56] van der Put NM, Gabreels F, Stevens EM, Smeitink JA, Trijbels FJ, et al (1998) A second common mutation in the methylenetetrahydrofolate reductase gene: an additional risk factor for neural-tube defects? Am J Hum Genet 62: 1044–1051. 9545395 10.1086/301825 PMC1377082

[57] O'Leary VB, Mills JL, Parle-McDermott A, Pangilinan F, Molloy AM, et al (2005) Screening for new MTHFR polymorphisms and NTD risk. Am J Med Genet A 138A: 99–106. 16145688 10.1002/ajmg.a.30846

